# Loss and resiliency of social amoeba symbiosis under simulated warming

**DOI:** 10.1002/ece3.6909

**Published:** 2020-10-20

**Authors:** Longfei Shu, Xinye Qian, Debra A. Brock, Katherine S. Geist, David C. Queller, Joan E. Strassmann

**Affiliations:** ^1^ Environmental Microbiomics Research Center School of Environmental Science and Engineering Southern Marine Science and Engineering Guangdong Laboratory (Zhuhai) Sun Yat‐sen University Guangzhou China; ^2^ Department of Biology Washington University in St. Louis St. Louis MO USA

**Keywords:** bacterial symbionts, *Dictyostelium discoideum*, global warming, *Paraburkholderia*, symbiosis

## Abstract

Anthropogenic global change is increasingly raising concerns about collapses of symbiotic interactions worldwide. Therefore, understanding how climate change affects symbioses remains a challenge and demands more study. Here, we look at how simulated warming affects the social ameba *Dictyostelium discoideum* and its relationship with its facultative bacterial symbionts, *Paraburkholderia hayleyella* and *Paraburkholderia agricolaris*. We cured and cross‐infected ameba hosts with different symbionts. We found that warming significantly decreased *D. discoideum's* fitness, and we found no sign of local adaptation in two wild populations. Experimental warming had complex effects on these symbioses with responses determined by both symbiont and host. Neither of these facultative symbionts increases its hosts’ thermal tolerance. The nearly obligate symbiont with a reduced genome, *P. hayleyella*, actually decreases *D. discoideum's* thermal tolerance and even causes symbiosis breakdown. Our study shows how facultative symbioses may have complex responses to global change.

## Graphical abstract

Experimental warming had complex effects on the social amoeba symbioses with responses determined by both symbiont and host. The less facultative symbiont, *P. hayleyella*, actually decreases *D. discoideum's* thermal tolerance and even causes symbiosis breakdown.

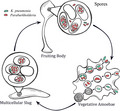

## 1 INTRODUCTION

Global warming causes biodiversity crises, which impact organisms not only directly but also indirectly through other organisms with which they interact (Bellard et al., 2012; Blois et al., 2013; Harley, 2011; Penuelas et al., 2013; Ullah et al., 2018). Symbiosis is important for global biodiversity, ecosystem services, and agriculture (Soka & Ritchie, 2015; Wernegreen, 2012; Werner et al., 2018). In recent years, the possibility that elevated temperatures resulting from global warming may substantially affect biodiversity through disrupting mutualistic associations such as the coral–dinoflagellate symbiosis (Hoegh‐Guldberg et al., 2007; Pandolfi et al., 2011), insect–bacteria symbioses (Kikuchi et al., 2016; Wernegreen, 2012), and plant–pollinator interactions (Eckert et al., 2010; Hegland et al., 2009) has been highlighted. The coral–dinoflagellate model, which is an obligate symbiotic relationship, showed that thermal stress could lead to coral bleaching (corals’ loss of zooxanthellae that provide up to 90% of host nutritional requirements) (Baker et al., 2018; Ferrier‐Pages et al., 2018). The stable, long‐term mutualistic relationship between insects and their carried symbionts is also vulnerable to thermal stress (Kiers et al., 2010). However, empirical investigations of facultative mutualism under global warming have been scarce and mostly focus on insects (Burke et al., 2010; Wernegreen, 2012). Research on facultative symbiosis is needed.

The symbiosis between social amebae and certain *Paraburkholderia* bacterial species is a promising system for gaining insight into how facultative mutualisms respond to global warming. The soil‐dwelling ameba *Dictyostelium discoideum* is a good model to address eukaryote–microbe interactions because of its dynamic relationship with bacteria. In a nutrient‐rich environment, *D. discoideum* lives as independent haploid amebae that reproduce by binary fission. When food is scarce, cAMP‐mediated aggregation occurs, leading to the formation of multicellular slugs that move to a favorable location to develop into fruiting bodies. In these fruiting bodies, approximately 20% of the cells die to form a long thin stalk that supports a spherical structure called the sorus, while the remaining 80% ascend into the sorus and turn into spores (Kessin, 2001). *D. discoideum* is a predator of bacteria and a popular model for studying biological phenomena, including multicellularity, chemical signaling, and social phenomena (Chen et al., 2016; DiSalvo et al., 2015; Ho et al., 2013; Kessin, 2001; Shu et al., 2018; Strassmann & Queller, 2011; Zhang et al., 2016).

**FIGURE 1:**
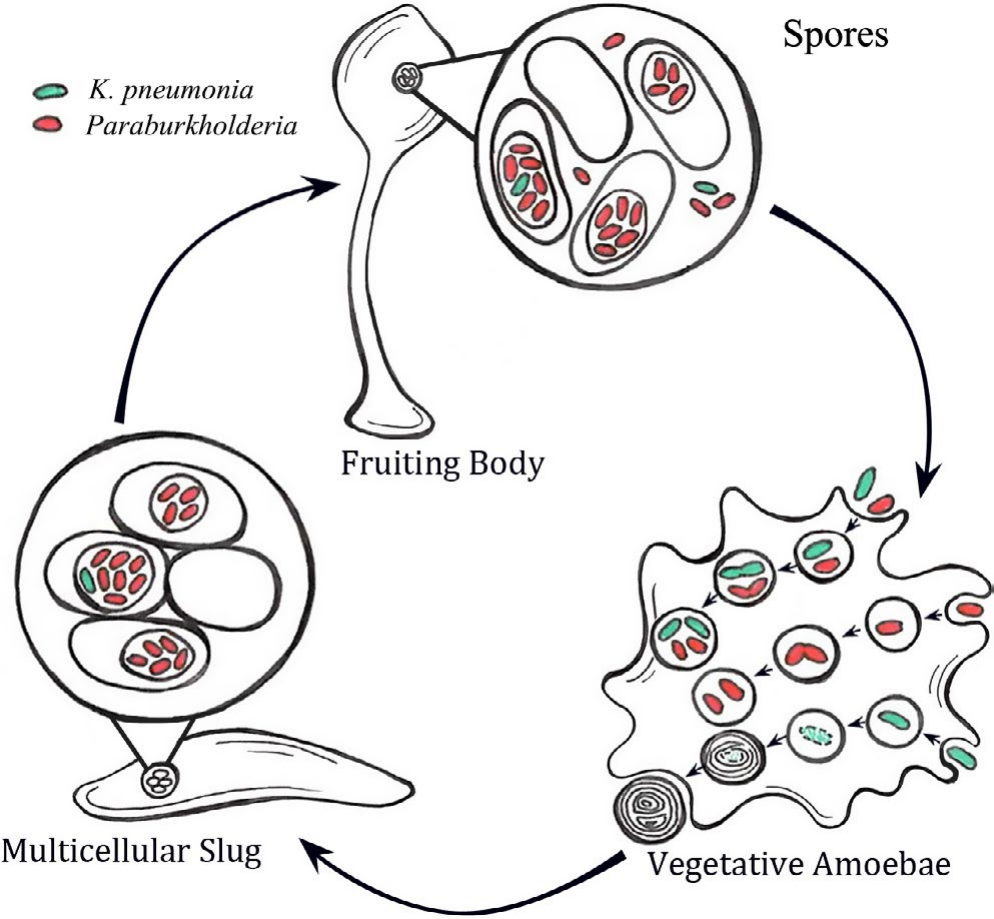
Scheme summarizing the social ameba–*Paraburkholderia* symbiosis. Figure courtesy of Susanne DiSalvo

In addition to eating bacteria, *D. discoideum* can also form symbiotic associations with some bacterial species (Brock et al., 2011; DiSalvo et al., 2015; Strassmann & Shu, 2017). About one‐third of wild‐collected clones of *D. discoideum*, which are referred to as “primitive farmers,” have stable associations with their symbiotic bacteria throughout their life cycle (Brock et al., 2011). These farmer clones can carry bacteria during spore dispersal and seed them as new food sources (Figure 1). Later studies found that farming status is induced by symbiotic bacteria belonging to the genus *Paraburkholderia* (DiSalvo et al., 2015; Haselkorn et al., 2019; Shu, et al., 2018) (named *P. agricolaris*, *P. hayleyella,* and *P. bonniea* (Brock et al., 2018)). These *Paraburkholderia* are not edible themselves, but they facilitate further carriage of food bacteria that on their own would be digested. The inedible symbionts actively find their ameba hosts through chemotaxis, reside within food vacuoles, and form very stable associations (Figure 1) (Shu et al., 2015; Haselkorn et al., 2019; Shu, et al., 2018; Shu, et al., 2018). Therefore, we also define their association as “bacterial carriage” by social ameba.

Both *D. discoideum* and their *Paraburkholderia* symbionts can live independently, making them facultative symbioses. However, *P. hayleyella* shows three indications of being more obligate than *P. agricolaris*. First, it is a sister species comprising a very long branch in the phylogeny, suggesting that it has been associated with amebas for a long time (Brock et al., 2018; Haselkorn et al., 2019). Second, consistent with greater dependence on the host, it grows slowly on its own under laboratory conditions compared to *P. agricolaris*. *P. hayleyella* also has greatly reduced carbon usage compared to *P. agricolaris* (Brock et al., 2020). Finally, it shows the genome size reduced by over one half compared to close relatives (Brock et al., 2018). This system gives us an opportunity to investigate how increased temperatures associated with global warming could potentially affect facultative symbioses.

**FIGURE 2:**
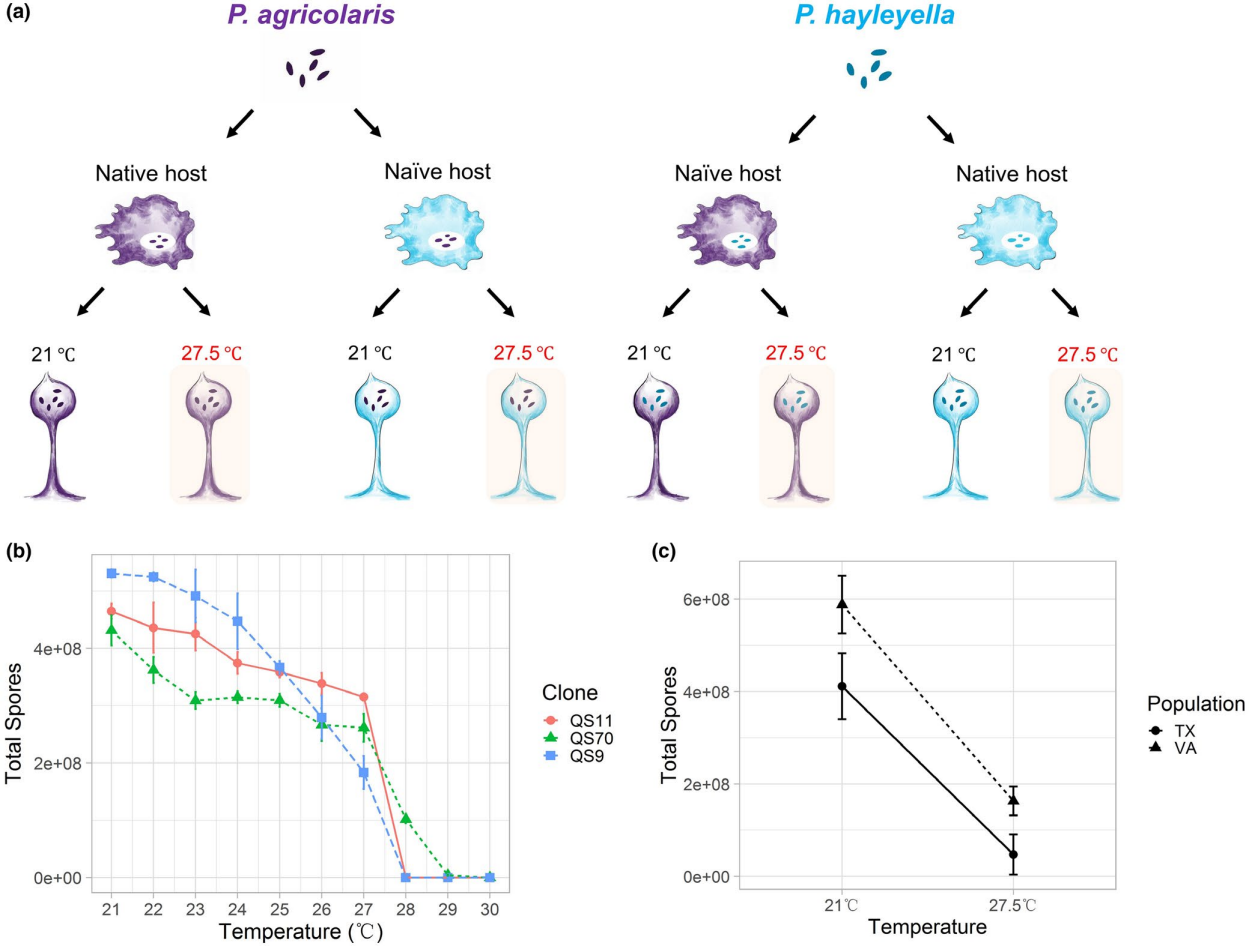
(a) Diagram of symbiosis experimental design. The experiment explores how thermal stress affects *D. discoideum*–*Paraburkholderia* symbiosis by mixing and matching *D. discoideum* with two facultative symbionts *P. agricolaris* and *P. hayleyella*. (b) Spore count (mean ± 95% CI) of three *D. discoideum* clones under different temperatures ranging from 21 to 30°C. QS9, naïve host; QS11, native host carrying *P. hayleyella* B2qs11, and QS70, native host carrying *P. agricolaris* B1qs11; (c) Spore count (mean ± 95% CI) of two *D. discoideum* populations (Texas and Virginia) under two temperature treatments (27.5 and 21°C). All tested Texas and Virginia clones are naïve host which do not carry any *Paraburkholderia* symbionts

Facultative symbioses could be more vulnerable to global warming compared to obligate symbioses because their relationships are less stable. Alternatively, facultative symbioses may be more resilient to global warming because both partners can live on their own and therefore may be more resilient to environmental changes. We will test whether these facultative symbionts help or harm their hosts under warming, and also whether the symbiosis is less or more resilient with the more facultative species *P. agricolaris* versus the more obligate species *P. hayleyella*. We first tested the thermal tolerance of social amebas using common garden experiments. Then, we mixed and matched social ameba hosts with different *Paraburkholderia* symbionts (Figure 2a) to investigate how different combinations respond to simulated global warming.

## 2 MATERIALS AND METHODS

### 2.1 *D. discoideum* clones and culture conditions

We used wild *D. discoideum* isolates (Table 1) collected at Mountain Lake Biological Station in Virginia (N37°21′, W80°31′), Houston Arboretum and Nature Center in Texas (N29°77′, W95°45′) and Little Butt's Gap in North Carolina (35°46′ N, 82°20′ W). These clones were uninfected (called naïve hosts in this paper) or infected with either *P. agricolaris* or *P. hayleyella* (called native hosts in this paper). We grew *D. discoideum* from previously frozen spores on SM/5 agar plates (2 g glucose, 2 g BactoPeptone (Oxoid), 2 g yeast extract (Oxoid), 0.2 g MgCl_2_, 1.9 g KH_2_PO_4_, 1 g K_2_HPO_4_, and 15 g agar per liter) with food bacterium *Klebsiella pneumoniae* (obtained from the Dicty Stock Center) at room temperature (21°C).

**TABLE 1:** List of wild *D. discoideum* clones and *Paraburkholderia* isolates used in this study. Checkmarks indicate specific clones in each test

Clones	Location	Host types	Symbionts	Choosing test temperature	Amebae under warming	Symbioses under warming
QS177	Texas	Naïve host			√	
QS198	Texas	Naïve host			√	
QS323	Texas	Naïve host			√	
QS325	Texas	Naïve host			√	
QS600	Texas	Naïve host			√	
QS68	Texas	Naïve host			√	
QS71	Texas	Naïve host			√	
QS74	Texas	Naïve host			√	√
QS76	Texas	Naïve host			√	
QS78	Texas	Naïve host			√	
QS1010	Virginia	Naïve host			√	
QS1041	Virginia	Naïve host			√	
QS1068	Virginia	Naïve host			√	
QS1072	Virginia	Naïve host			√	
QS1080	Virginia	Naïve host			√	
QS17	Virginia	Naïve host			√	
QS18	Virginia	Naïve host			√	
QS4	Virginia	Naïve host			√	
QS6	Virginia	Naïve host			√	
QS9	Virginia	Naïve host		√	√	√
QS1	Virginia	Naïve host				√
QS70	Virginia	Native host	*P. agricolaris* B1qs70	√		√
QS159	Virginia	Native host	*P. agricolaris* B1qs159			√
NC21	North Carolina	Native host	*P. agricolaris* B1nc21			√
QS11	Virginia	Native host	*P. hayleyella* B2qs11	√		√
QS21	Virginia	Native host	*P. hayleyella* B2qs21			√
NC28	North Carolina	Native host	*P. hayleyella* B2nc28			√
QS70C	Virginia	Cured native host				√
QS159C	Virginia	Cured native host				√
NC21C	North Carolina	Cured native host				√
QS11C	Virginia	Cured native host				√
QS21C	Virginia	Cured native host				√
NC28C	North Carolina	Cured native host				√

### 2.2 Symbionts

We used *D. discoideum*‐associated *Paraburkholderia* symbionts isolated and described in previous studies (Brock et al., 2011; DiSalvo et al., 2015; Haselkorn et al., 2019; Shu, et al., 2018). *P. agricolaris* strains were isolated from QS70, QS159, and NC21 *D. discoideum* hosts, while *P. hayleyella* strains were isolated from QS11, QS21, and NC28 *D. discoideum* hosts, respectively. Specific isolates used in this study are listed in Table 1.

### 2.3 Choosing experimental temperature for simulating warming

We wanted to choose an experimental temperature that is stressful to social amebae but does not cause complete death. We tested growth conditions of *D. discoideum* (three clones: QS11, QS70, and QS9) under different temperatures ranging from 21 to 30°C. We found that almost no clone can survive above 28°C, while there were drastic changes between 27 and 28°C (Figure 2b). Therefore, we chose 27.5°C as the thermal stress temperature for this experiment. We want to test how extreme warming event (from *D. discoideum* ameba's perspective) affects the social ameba symbiosis and whether its bacterial symbionts could help.

### 2.4 Effects of thermal stress on two wild *D. discoideum* populations

We used two *D. discoideum* populations from geographic and climate divergent locations Texas (N29°46′, W95°27′; elevation, 11 m; annual temperatures: 5.7–34.7°C; average temperatures: 20.6°C) and Virginia (N37°21′, W80°31′; elevation, 1,160 m; annual temperatures: −15–25°C; average temperatures: 5.2°C) to investigate how *D. discoideum* responds to simulated thermal stress and whether they could locally adapt to it. We randomly chose 10 Texas clones and 10 Virginia clones of wild *D. discoideum* and plated those (2 × 10^5^ spores) in association with *K. pneumoniae* (200 µl, OD1.5) on SM/5 plates. We incubated these clones at room temperature 21°C (control) and 27.5°C (thermal stress treatment), respectively. We harvested spores from each plate after one week. We flooded the plate with 10 ml KK2 + 0.1%NP‐40 and collected spores into 15 ml falcon tubes. We counted spores on a hemocytometer using a light microscope. This design resulted in a total of 2 (populations) × 10 (clones) × 2 (temperatures) × 3 (replicates) =  120 experimental units. The mean of three replicates was used for further statistical analyses.

### 2.5 Effects of thermal stress on *D. discoideum*–*Paraburkholderia* symbiosis

We generated symbiont‐free native host clones (QS70C, QS159C, NC21C, QS11C, QS21C, and NC28C) by curing them of their bacteria with tetracycline, or by ampicillin–streptomycin treatment as previously described (Brock et al., 2011; DiSalvo et al., 2015; Shu, et al., 2018). We confirmed the loss of infection status by plating them out on bacteria‐free plates and confirming that the social amebae could not proliferate, a test we call a spot test (Brock et al., 2011).

We mixed and matched (Figure 2) *D. discoideum* (naïve hosts: QS1, QS9, and QS74; native hosts: QS70C, QS159C, NC21C, QS11C, QS21C, and NC28C) with two facultative symbionts *P. agricolaris* (B1qs70, B1qs159, and B1nc21) and *P. hayleyella* (B2qs11, B2qs21, and B2nc28) to investigate how thermal stress affects their symbiotic relationships. We tested four combinations under two temperature treatments (21 and 27.5°C): native hosts—*P. agricolaris*, naïve hosts—*P. agricolaris*, native hosts—*P. hayleyella* and naïve hosts—*P. hayleyella* with three replicates.

To set up each experiment, we plated 2 × 10^5^ spores in association with *K. pneumoniae* (200 µl, OD1.5) on SM/5 plates. For experiments adding *Paraburkholderia*, we mixed the specified *Paraburkholderia* (OD1.5) clones at 3% (6 µl) and *K. pneumoniae* at 97% (194 µl) vol and plated *D. discoideum* spores (2 × 10^5^) with 200 µl of the bacterial mixture on SM/5 plates. We incubated these clones at room temperature 21°C (control) and 27.5°C (thermal stress treatment), respectively. We harvested spores from each plate after one week and flooded the plate with 10 ml KK2 + 0.1%NP‐40 and collected spores into 15 ml falcon tubes. We counted spores on a hemocytometer using a light microscope.

### 2.6 Statistical analyses

#### 2.6.1 Effects of thermal stress on two *D. discoideum* populations

We analyzed the data (*N* = 40) with a general linear mixed model in IBM SPSS Statistics 24. In these analyses, population (two levels: Texas and Virginia), temperature (two levels: 21 and 27.5°C), and their interactions were used as fixed factors. *D. discoideum* clone was nested within population and used as a random factor. The data passed the normality test (Kolmogorov–Smirnov test) and tested for homogeneity of variance (Levene's test).

We analyzed spore production (outcome variable) as a measure of ameba fitness. A significant temperature main effect would indicate thermal stress affects *D. discoideum's* fitness, a significant population type main effect would indicate that populations differ in their fitness, and a significant population × temperature interaction would indicate adaptive divergence in thermal tolerance in two populations.

#### 2.6.2 Effects of thermal stress on *D. discoideum*–Paraburkholderia symbiosis

**FIGURE 3:**
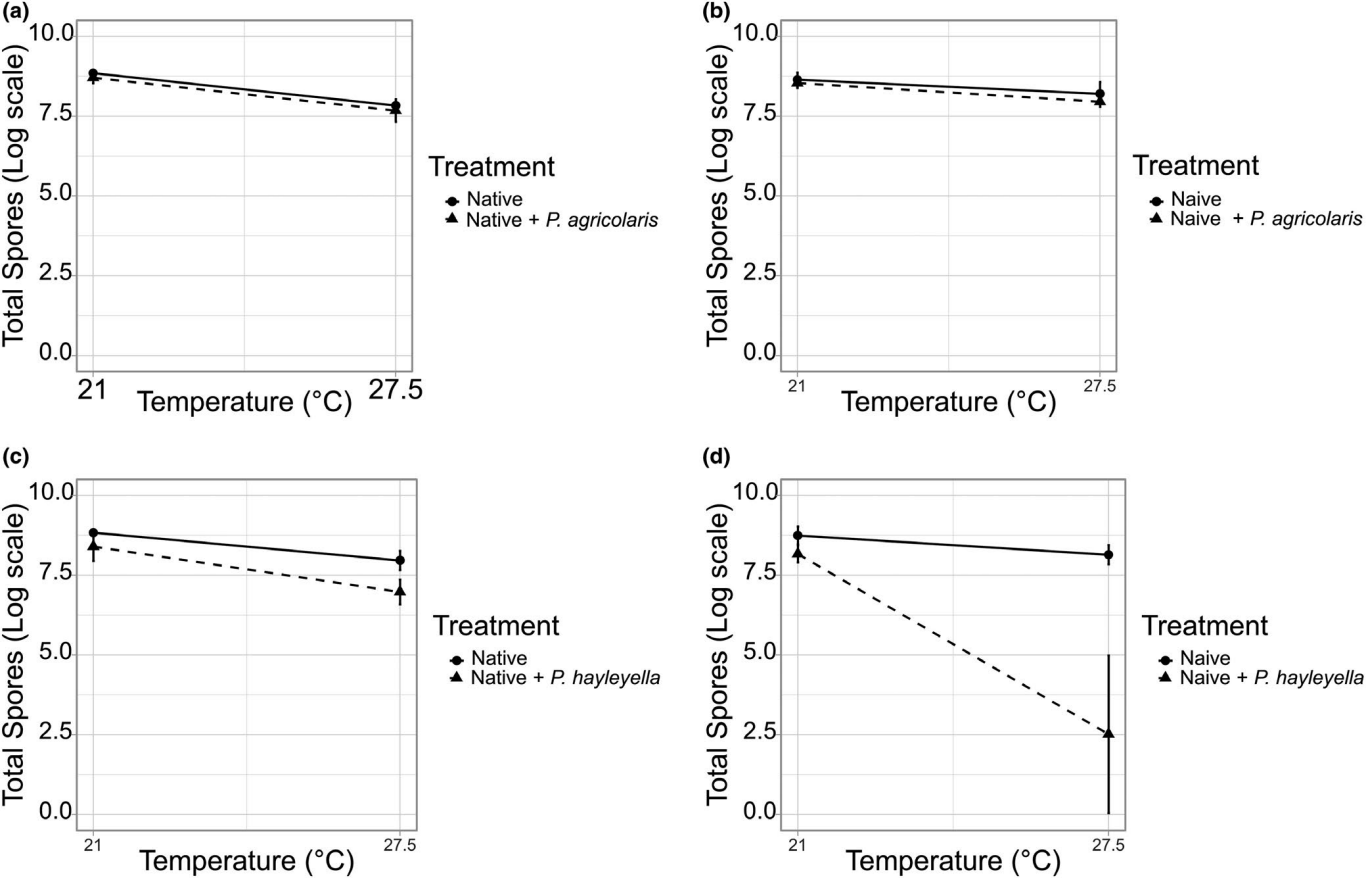
Spore counts (mean ± 95% CI) of *D. discoideum* hosts (with and without *P. agricolaris* and *P. hayleyella*) under two temperature treatments (27.5 and 21°C). (a) *P. agricolaris* with their native hosts (hosts which they are isolated: QS70, QS159, NC21); (b) *P. agricolaris* with naïve hosts (non‐farmer clones: QS1, QS9, QS74); (c) *P. hayleyella* with their native hosts (hosts which they are isolated: QS11, QS21, NC28); (d) *P. hayleyella* with naïve hosts (non‐farmer clones: QS1, QS9, QS74)

We analyzed and plotted four combinations separately (native hosts—*P. agricolaris*, Figure 3a; naïve hosts—*P. agricolaris*, Figure 3b; native hosts—*P. hayleyella,* Figure 3c and naïve hosts—*P. hayleyella,* Figure 3d). Native hosts—*P. agricolaris* (*N* = 12), naïve hosts—*P. agricolaris* (*N* = 24), and native hosts—*P. hayleyella* (*N* = 12) data were log‐transformed to improve normality. Transformed data passed the normality test (Kolmogorov–Smirnov test) and tested for homogeneity of variance (Levene's test). We analyzed these data with general linear models. Naïve *P. hayleyella* data (*N* = 24) were analyzed with a generalized linear model (GLM) with Negative binomial distribution in IBM SPSS Statistics 24.

We used spore production as a measure of ameba fitness. A significant temperature main effect indicates that thermal stress can affect *D. discoideum* fitness. A significant symbiont main effect indicates that the presence of a symbiont can affect *D. discoideum* fitness. A significant temperature × symbiont interaction will indicate that the presence of symbiont can affect *D. discoideum* fitness under thermal stress.

## 3 RESULTS

### 3.1 The pattern of local adaptation to thermal stress in *D. discoideum*

Increased temperature decreased the fitness of both the Texas clones and the Virginia clones (Figure 2c), as indicated by the significant temperature main effect (GLM, *F*_1,18_ = 351.25, *P* < .001). Virginia clones outperformed Texas clones at both temperatures (Figure 2c). However, we found no variation in thermal tolerances of Texas and Virginia populations, as shown by the nonsignificant population × temperature interaction (GLM, *F*_1,18_ = 2.141, *P* = .161). These results suggest that increased temperature significantly decreases *D. discoideum's* fitness. We did not find adaptive divergence to thermal stress in two wild populations of *D. discoideum* from locations that differed in ambient temperature.

### 3.2 The complex effects of simulated warming on *D. discoideum*–*Paraburkholderia* symbioses

#### 3.2.1 *P. agricolaris* had no effect on *D. discoideum's* thermal tolerance

When *P. agricolaris* clones were mixed with their native hosts, thermal stress decreased *D. discoideum's* fitness, as indicated by the significant temperature main effect (GLM, *F*_1,20_ = 20.188, *p* < .001, Figure 3a). However, adding *P. agricolaris* made no difference to host fitness (GLM, *F*_1,20_ = 2.406, *p* = .137, Figure 3a). The effect of thermal stress did not change with the addition of *P. agricolaris*, as indicated by the nonsignificant temperature*symbiont interaction (GLM, *F*_1,20_ = 0.427, *p* = .521, Figure 3a).

When *P. agricolaris* clones (*n* = 3) were mixed with naïve hosts (*n* = 3), the pattern is the same (Figure 3b). Thermal stress decreased *D. discoideum's* fitness (General linear model, *F*_1,8_ = 82.087, *p* < .001, Figure 3b), while adding *P. agricolaris* made no difference to host's fitness (GLM, *F*_1,8_ = 1.803, *p* = .216, Figure 3b). Also, there was no significant temperature*symbiont interaction (General linear model, *F*_1,8_ = 0.004, *p* = .953, Figure 3b), indicating that the effect of thermal stress did not change with the addition of *P. agricolaris*.

Overall, these results suggest that the more facultative *P. agricolaris* neither helps nor harms *D. discoideum* under thermal stress. In addition, there is no difference between native and naïve hosts.

#### 3.2.2 *P. hayleyella* decreased *D. discoideum's* thermal tolerance and caused a symbiosis breakdown when mixed with naïve hosts

When *P. hayleyella* clones (*n* = 3) were mixed with their native hosts (*n* = 3), thermal stress decreased *D. discoideum's* fitness, as indicated by the significant temperature main effect (GLM, *F*_1,8_ = 44.747, *p* < .001, Figure 3c). We also found that adding *P. hayleyella* decreased host fitness (GLM, *F*_1,8_ = 17.287, *p* = .003, Figure 3c). There was no significant temperature*symbiont interaction (GLM, *F*_1,8_ = 2.624, *p* = .144, Figure 3c), indicating that adding *P. hayleyella* did not further decrease the native host's fitness under thermal stress (Figure 3c).

When *P. hayleyella* clones (*n* = 3) were mixed with naïve hosts (*n* = 3), both adding *P. hayleyella* (Negative binomial GLM, χ^2^ = 6.73, *p* = .009) and thermal stress (Negative binomial GLM, χ^2^ = 8.471, *p* = .004) decreased *D. discoideum's* fitness (Figure 3d). There was also a significant temperature*symbiont interaction (Negative binomial GLM, χ^2^ = 4.958, *p* = .026, Figure 3d), indicating that adding *P. hayleyella* further decreased naïve host's fitness under thermal stress. In addition, 2 out of 3 tested naïve hosts showed zero growth under thermal stress when mixed with *P. hayleyella*, indicating symbiosis breakdown, while this did not happen in any of the native hosts.

Taken together, these results suggest that adding *P. hayleyella*, like thermal stress, can decrease *D. discoideum's* fitness. In addition, it further decreases host fitness under thermal stress. We also found evidence of symbiosis breakdown when *P. hayleyella* was mixed with naïve hosts, while this does not happen in the native hosts. This indicates potential partner adaptation between *P. hayleyella* and their native hosts.

## 4 DISCUSSION

Overall, we show that increased temperature affects symbiotic interactions. Increased temperature can significantly decrease *D. discoideum's* fitness. We found no adaptive divergence to thermal stress in two wild populations. Neither symbiont increased its hosts’ thermal tolerance. Our study shows that facultative symbioses can also have complex responses to warming.

Previous studies found that facultative symbionts provide greater flexibility in response to temperature change compared to obligate symbioses (Burke et al., 2010; Renoz et al., 2019). For example, facultative bacterial symbionts benefit aphids under heat stress (Montllor et al., 2002) and may protect both host and obligate symbiont from thermal stress (Burke et al., 2010). However, in the social ameba symbiosis system, we find no evidence that facultative *Paraburkholderia* symbionts increase *D. discoideum* hosts’ thermal tolerance.

We find that different symbionts behave differently within the same host under simulated warming, and we also find evidence of host adaptation. Of the two symbionts, the more facultative *P. agricolaris* has no effects on the thermal tolerance of either native or naïve *D. discoideum* hosts. On the other hand, the more obligate *P. hayleyella* induces a significant difference to the host's thermal tolerance, imposing a higher cost to *D. discoideum*. Our study shows that the addition of *P. hayleyella* to its native host decreases host fitness at both temperatures indicating that native hosts suffer a fitness cost when they carry *P. hayleyella*. In addition, *P. hayleyella* harms and even kills naïve hosts exposed to thermal stress, disrupting the symbiosis. The more severe fitness costs exerted by *P. hayleyella* colonization in naïve hosts compared to native hosts suggest potential host adaptation between *P. hayleyella* and their native host clones.

One potential drawback of this study is that we did not monitor the population dynamics of *K. pneumoniae* and *Paraburkholderia* symbionts under different temperatures. Simulated warming can directly affect the interactions between food bacteria and symbionts, which in turn affects the growth of amebae. Indeed, a recent study reported that the optimal growth temperature for both *Paraburkholderia* symbionts is 30°C, and *P. agricolaris* grows faster than *P. hayleyella* (Brock et al., 2020). Therefore, in this study, both food bacterium *Klebsiella pneumoniae* and *Paraburkholderia* symbionts grow faster under warming conditions. However, we argue that their interactions may have little effect on host fitness. First, *K. pneumoniae* grows much faster than symbionts, and their starting proportion is very high (97%) compared to symbionts (3%). Second, the faster‐growing symbiont, *P. agricolaris*, did not change host's fitness in both temperatures, indicating its frequency has little effect on host fitness. Moreover, *P. hayleyella* grows much slower than *P. agricolaris*. Therefore, despite their faster growth under warmer temperatures, the major conclusion of this study still holds. Still, it will be useful to have such information in future studies.

Taken together, our results provide insight into facultative symbioses under extreme warming. For the ameba–*Paraburkholderia* symbiotic relationship, the effects of adding different *Paraburkholderia* can be complex. The responses of social ameba symbioses to warming depend on both symbiont types and host types. Our study also shows that facultative symbionts are not necessarily more resilient to global change. In this system, the less facultative, more obligate symbiont has the less resilient symbiosis. Different symbioses may develop different evolutionary trajectories leading to unpredictable symbiosis resiliency with global warming.

## CONFLICT OF INTEREST

The authors declare no conflicts of interest.

## AUTHOR CONTRIBUTION

**Longfei Shu:** Conceptualization (equal); Data curation (equal); Formal analysis (equal); Funding acquisition (equal); Visualization (lead); Writing‐original draft (lead). **Xinye Qian:** Data curation (equal); Formal analysis (equal); Visualization (equal); Writing‐original draft (equal). **Debra A. Brock:** Data curation (equal); Formal analysis (equal); Writing‐review & editing (equal). **Katherine S. Geist:** Data curation (equal); Formal analysis (equal); Visualization (equal); Writing‐review & editing (equal). **David C. Queller:** Conceptualization (lead); Data curation (equal); Formal analysis (equal); Funding acquisition (lead); Supervision (lead); Writing‐review & editing (equal). **Joan E. Strassmann:** Conceptualization (lead); Data curation (equal); Formal analysis (equal); Funding acquisition (lead); Supervision (lead); Writing‐original draft (equal); Writing‐review & editing (equal).

## Data Availability

All data are available from the Mendeley Data: http://dx.doi.org/10.17632/fjj9mbm6hw.1
